# Can Judgments of Threat Reflect an Approaching Person’s Trait Aggression?

**DOI:** 10.1007/s12144-016-9557-5

**Published:** 2017-01-07

**Authors:** Liam Satchell, Paul Morris, Lucy Akehurst, Ed Morrison

**Affiliations:** 1 Centre for Situated Action and Communication, Department of Psychology, University of Portsmouth, King Henry Building, King Henry 1 Street, Portsmouth, PO1 2DY UK; 2 International Centre for Research in Forensic Psychology, Department of Psychology, University of Portsmouth, King Henry Building, King Henry 1 Street, Portsmouth, PO1 2DY UK; 3 The Centre for Comparative and Evolutionary Psychology, Department of Psychology, University of Portsmouth, King Henry Building, King Henry 1 Street, Portsmouth, PO1 2DY UK

**Keywords:** Threat perception, Gait behaviour, Trait aggression

## Abstract

When in a vulnerable situation (such as walking alone at night), an approaching person may be seen as ‘threatening’. Here, we are interested in how well participants’ judgments of threat reflected the trait aggression of approaching target people. We use two similar experiments to demonstrate and replicate the relationship between judgments of threat and target aggression.

In both studies participants judged how threatening they found 22 approaching people (presented in videos). In Study One, participants judged the targets whilst sitting at a computer. In Study Two, participants were standing and were either oriented facing the videos, or oriented away from the videos so they had to look over their shoulder. This was to emulate a potentially threatening person approaching from behind. Across both studies, there was strong evidence that the average judgments of the threat posed by the approaching targets accurately reflected the targets’ trait aggression. It was also found that there was noteworthy variability in individual participants’ ability to detect aggression, with a few participants even having an inverse relationship between threat and the target’s aggression. This research demonstrates that judgments of how ‘threatening’ a person is can be used to accurately index trait aggression at a distance.

When walking down a street in a vulnerable situation (such as alone at night), an approaching stranger could be seen as a threat to personal safety. Typically people act on feeling threatened and engage in avoidance behaviours (changes in direction or own posture). Failure to recognise a genuinely aggressive person as a threat, and the subsequent failure to make an avoidance response, could result in injury or worse. This judgment is also time sensitive: as another person approaches, the time to make and act on a threat judgment becomes increasingly limited. Thus, it is of interest to explore judgments of threat made when observing an approaching person. It will also be important to test if these judgments of threat are useful: can threat judgments reflect the dispositional aggression of an approaching person? In two studies we will investigate *i*) can judgments of the threat posed by an approaching person reflect that person’s trait aggression? And *ii*) are judgments of the threat posed by an approaching person still accurate when observing someone who would appear to be approaching from behind?

There is a large literature that shows that, when viewing photographs of a target person’s face, people can make accurate judgments of how aggressive another person is (Boshyan et al. 2013; Carré et al. 2009; Carré et al. 2010) and a target person’s fighting ability (Sell et al. 2009). The ability to understand another person’s disposition from briefly observing a face is a useful skill as it can allow for an individual to successfully avoid a potential danger. However, these opportunities to avoid an approaching person tend to occur at a distance, allowing someone to avoid a threatening person before they are too close. The optimal distance for avoiding another person could occur before many of the details of the face are available. The whole body is the most typical way that individuals encounter others in the world (Azarian et al. 2015) and this is highlighted when making judgments of a person approaching from a distance. Other information about a person may be particularly available at a distance. For example, body size (Deaner et al. 2012) and gait style (Satchell et al. 2016) have been shown to also predict tendencies towards aggression and could inform judgments of threat. Therefore we choose to use presentations of approaching people that include street-typical information that would be available to someone making an everyday threat judgment. We present our participants (henceforth referred to as ‘Judges’) with videos of approaching people which include the target’s faces (Boshyan et al. 2013; Carré et al. 2009; Carré et al. 2010), body shape (Deaner et al. 2012) and gait style (Satchell et al. 2016), all of which may help a participant reach an accurate threat judgment.

Other researchers have highlighted how varying the amount of information available to a perceiver can influence interpersonal judgment accuracy. The Realistic Accuracy Model (RAM; Funder 1999) is a model of personality judgment that describes how judgments of other people can be accurate. The RAM has four parts and suggests that; when a target’s behaviours, *Relevant* to the trait being judged, are *Available* for a judge to *Detect,* then the judge can correctly *Utilize* the information to form an accurate perception. If we apply the RAM to existing research, we can see most studies have been limiting the *Available* information for judges by using photos of faces. Given the research showing that there is more *Relevant* information for judging aggression than face shape (i.e., gait and body shape), it is possible that threat judgments could be particularly strong predictors of aggression when an approaching person is viewed in a more street-typical context. We do recognise that videos of an approaching person do not replicate the precise amount of information that would be available in real world environments (and the context for making judgments is very different). However, we consider this move towards more street-typical presentations of potential dangerous others important for our understanding of this research in its everyday context.

We report two studies here which present 22 videos of approaching targets to judges. The first study is a ‘proof of concept’ study, investigating the ability of threat judgments to reflect aggression when judges are exposed to ‘street-judgment typical’ stimuli. The second study addresses a real world question: what happens if the target approaches from behind the judge? The second experiment uses two conditions where judges are standing and are either ‘oriented towards’ (facing) the approaching targets or ‘oriented away’ from the targets. In the oriented away condition, judges looked over their shoulder at the targets, as if the person was approaching from behind. Looking to the literature, we could suggest that judgments of threat could be affected by the nature of this ‘facing away’ from the targets. Previous research has demonstrated that disliking or avoidance of others can be manifest by turning away from that person (McCall et al. 2009; McCall and Singer 2015) so perhaps, through the embodiment of being oriented towards or away from a target of threat ratings, threat judgments could be affected. This prediction is exploratory, and our main reason for the manipulation is to continue to move towards a street-typical setting in our laboratory research.

In both studies we report both “nomothetic” (Kolar et al. 1996, p.321) and “idiographic” (Kolar et al. 1996, p.326) analyses of our data (as recommended by; Brand and Bradley 2012; Kolar et al. 1996; Monin and Oppenheimer 2005) to reveal how judges generally perform in the task (sample mean), as well as describing the individual judges’ performance. Idiographic analyses limit the error from using aggregated variables (the product of a whole sample’s data) in the correlation by computing a correlation for each judge and then reporting the distribution of the judges’ performance. In both studies we predict that there will be general accuracy; threat judgments will reflect targets’ aggression.

The two studies here use arguments from person judgment theories (RAM; Funder 1999) to build on the previous robust research on accurately judging the malevolent traits of another person (Boshyan et al. 2013; Carré et al. 2009; Carré et al. 2010) by making street-typical information *Available* to judges, such as body shape (Deaner et al. 2012) and gait style (Satchell et al. 2016).

## Study One: Do Judgments of Threat Reflect the Aggression of an Approaching Person?

Study One investigates the relationship between judges’ judgments of the threat posed by approaching targets and the targets’ self-reported trait aggression. This study was conducted using the standard lab paradigm, frequently used in the psychology literature, where judges are seated at a computer (akin to: Carré et al. 2009; Carré et al. 2010; Sell et al. 2009). We use this study as our first investigation of judge accuracy when presented with videos of approaching targets and therefore more *Available* (Funder 1999) information than has previously been used in similar studies.

## Method

### Participants

The sample were 61 undergraduate student judges (Female = 47, *M*_*Age*_ = 19.18 years, *SD*_*Age*_ = 3.34 years) were recruited from a participant pool at a UK university. They were compensated for their participation with course credit.

### Materials

The targets in this experiment were 22 individuals (Female = 11, Target *M*_Age_ = 20.50 years, *SD*_Age_ = 2.04 years) who were recorded walking on a treadmill at their chosen speed (so as to be a close to typical gait as possible). The targets were oriented towards the camera and a video camera recorded 10 s of uninterrupted gait. Targets wore standardised clothes; for male targets a white t shirt and blue shorts and for female targets a grey vest top and black leggings.

We opted for a self-report measure of aggression due to the noted concerns with the validity of laboratory measures of aggression (for a review see; Ritter and Eslea 2005; Tedeschi and Quigley 1996). The paradigms used in these studies do not reflect trait aggression and, in fact, these tasks also lack standardisation and reliability between studies (Elson et al. 2014). There are benefits of targets being able to use their own experiences to inform a self-report. As a measure of trait aggression, targets completed the Buss-Perry Aggression Questionnaire (Buss and Perry 1992, analysed using revisions suggested by Bryant and Smith 2001). This self-report measure has been shown to be valid in measuring hypothetical (Archer and Webb 2006; O’Connor et al. 2001) and historic (Diamond 2006) aggression, has strong reliability (Webster et al. 2013) and is frequently used in contemporary research to report trait aggression (such as Lake et al. 2014, Waldron et al. 2015; Zajenkowski and Zajenkowska 2015). We specifically use the physical aggression subscale for analysis as it was most relevant to detecting the likelihood of a target to physically assault a judge. As per Bryant and Smith’s (2001) revisions targets could score between 3 and 21 for the physical aggression measure. It is important to note that these young adults were not violent offenders but there was sufficient variation to allow for analysis of individual differences (*M*_Aggression_ = 7.46, *SD*_Aggression_ = 4.78, *Min*_Aggression_ = 3, *Max*_Aggression_ = 19).

### Procedure

Judges gave written informed consent. Judges took part in the experiment while seated at a computer and rated the videos of 22 targets on a scale of *Threatening (9) – Unthreat*ening (*1*) as well as distractor scales (which are not analysed here). The order of target presentation was randomised for each judge.

We note that this is different from the typical personality judgment paradigm, as we ask judges for their judgment of ‘threat’ rather than a direct judgment of the trait being assessed: Aggression. We do this because it is more typical (in everyday street judgments) to appraise the ‘threat’ posed by a target than how ‘aggressive’ that target is. As such, the instruction of judging ‘threat’ better fits this study’s objective of more naturalistic judgments of the targets.

### Analyses

For all analyses we report the relationship between threat judgments and trait aggression for just the male targets (*k* = 11), just the female targets (*k* = 11) and for all targets together (*K* = 22).

We analysed the judgment data in two ways. Firstly we report ‘nomothetic’ (Kolar et al. 1996) correlations, where we took the mean threat rating received by the targets (i.e. using the data from all judges for a single target) and correlated that with the targets’ self-reported trait aggression scores. Whilst this “nomothetic” (Kolar et al. 1996, p.321) style of analysis is typical for the literature, we also use “idiographic” (Kolar et al. 1996, p.326) analysis of judge accuracy (such as; Hirschmüller et al. 2015; Kolar et al. 1996), where each judge receives an accuracy correlation. A single judge’s threat judgments of all targets is correlated with the traits of all the targets. This gives judge *x* their own Pearson’s *r* value that acts as an expression of their accuracy (*r* = 1 being linear accuracy [where threat predicts aggression], *r* = −1 being linear inaccuracy [where threat is inverse to aggression], and *r* = 0 being random performance [no relationship between threat and aggression]). The process is then repeated for all judges. This allowed the reporting of the distribution of judges’ individual relationship between threat ratings and target aggression. The accuracy of any judge who did not show variance in their ratings of threat (e.g. all targets have Threat =1) has their accuracy considered as *r* = 0. This could be tested for significant performance above chance performance using a one-sample *t* test (see; Hirschmüller et al. 2015).

## Results

### How did the Average Threat Judgments Relate to the Targets’ Aggression?

Here, we report the standard nomothetic (see *Analyses* above; Kolar et al. 1996) analyses. There were notable positive correlations between the average threat rating received by the targets (the mean threat rating from the whole sample) and the targets’ trait aggression for the male targets (*r*(11) = .61, *95% CI* [.08, .87], *p* = .047) and the female targets (*r*(11) = .44, *95% CI* [−.12, .81], *p* = .181). Although the correlation is stronger for the male targets, the two correlations are not significantly different (Fisher’s *Z* = .47, *p* = .638) and the correlation for all targets together was moderate-large (*r*(22) = .45, *95% CI* [.09, .73], *p* = .035). These results show that judgments of the threat posed by approaching men and women can be used to detect trait aggression, although accuracy is better with just male targets.

### How did the Individual Judges’ Threat Judgments Relate to the Targets’ Aggression?

*W*e can also report individual accuracy correlations for each judge (see *Analyses*) and then test the judges’ accuracy against random performance (*r* = 0) in a one-sample t test (see; Hirschmüller et al. 2015). The distribution of judges’ accuracy can be seen in Table 1. All distributions have a positive mean (on average judges’ ratings of threat reflected the targets’ trait aggression) and that there was an interesting range of accuracy, that is to say, some judges had an inverse relationship between threat judgments and the targets’ trait aggression (*more* aggressive targets were judged as *less* threatening). When tested using a one-sample t test, the accuracy in judging male targets (*t*(60) = 7.27, *p* < .001, *d* = .93), female targets (*t*(60) = 3.50, *p* = .001, *d* = .45) and all targets together (*t*(60) = 8.17, *p* < .001, *d* = 1.05) were all notably above chance performance (*r* = 0).

**Table 1:** The distribution of idiographic accuracy correlations for the judges in Study One for male, female and all targets. (*N* = 61)

Targets	Mean	D	Minimum	Maximum	Skewness	Kurtosis
Male Targets Only (k = 11)	.28	.30	-.61	.81	-.75	.19
Female Targets Only (k = 11)	.13	.29	-.37	.78	.57	-.35
All Targets Together (k = 22)	.20	.19	-.30	.64	-.37	.22

## Study One Summary Discussion.

These results show that judgments of the threat posed by an approaching person reflects that person’s trait aggression. Some of the nomothetic accuracy (i.e. for male targets) and idiographic accuracy (i.e. the maximum scores in Table 1) correlations were notably strong, perhaps a product of increasing the *Available* (Funder 1999) information for judges in this study, compared to a face-only presentation of targets. Reporting idiographic results revealed that some judges were highly inaccurate at this task, seeing the more aggressive targets as *less* threatening (see Table 1). Inaccurate judgments of threat in everyday life can have consequences and as such those individuals who have lower accuracy or even inaccuracy are of as much interest as those who performed well in the task.

## Study Two: does Orientation toward the Target Affect Judgment Accuracy?

The results of the first study suggested that judgments of the threat posed by a video of an approaching person relates to the targets’ trait aggression. In the second study we explore the effect of viewing the video at a distance, as would happen in street-typical contexts. Furthermore, we look at the effect of the participant looking over their shoulder to look at the video, as people may look behind them to assess whether a person walking behind them is threatening. This question is an important step towards everyday threat judgments. There is also some evidence that the orientation of a judge, relative to a target, can reflect liking (see; McCall et al. 2009; McCall and Singer 2015). Although it is unclear if this disliking effect would impact liking of a target if manipulated in an experimental setting.

## Method

### Participants

A new sample of 58 undergraduate students (Female = 46, *M*_Age_ = 18.47 years, *SD*_Age_ = .88 years) took part in the experiment for a course credit. Judges were informed that they would be taking part in an experiment on interpersonal perception called “They’re Behind You?”

### Materials

The target videos for this study were the same as those used in Study One. Judges reported that the videos in Study One were longer than necessary for their judgments, so the videos were trimmed to use only the first 5 s of the videos in Study One. In this study the videos were projected onto a larger screen, see Procedure below.

### Procedure

Judges were randomly allocated to either the ‘towards’ or ‘away’ condition (*N* = 29 for each condition). Judges were standing 4 m away from a presentation screen and judged the targets on the same *Threatening (9) – Unthreat*ening (*1*) dimension ( as well as the distractor items (not analysed here). Judges were positioned in ‘footprints’, with their feet between wooden blocks (15 cm apart) so that they were either facing directly ‘towards’ the presentation of the targets or 120° away from the screen. Judges in the ‘away’ condition were instructed to keep their body aligned with their feet and to look over their shoulder at the stimuli. All judges self-reported no history of neck or back problems so could perform the instruction without issue. Judges completed the experiment in a room alone, but were observed by an experimenter (through a one-way window) to ensure that they remained in the footprints. The 22 targets were presented in a random order and judges were allowed as much time as they needed to complete their ratings before using a presentation remote to move on to the next video. Judges used a clipboard, pen and paper to make their ratings.

## Results

### Did the Orientation Conditions differ?

Contrary to our predictions, there was no difference in the accuracy of threat judgments in the oriented towards or away conditions. This was evident using the difference between conditions in judges’ idiographic accuracy scores (their individual Pearson *r* values, see Study One) for only male targets (*t*(56) = 0.97, *p* = .336, *d* = .26), only female targets (*t*(56) = 0.56, *p* = .575, *d* = .15) and all the targets together (*t*(56) = 0.19, *p* = .848, *d* = .05). Given that our manipulation did not elicit any differences in judgment accuracy, the data was combined for both conditions. Descriptive statistics for the overall sample, as well as for each of the two conditions, can be found in Table 2.

**Table 2:** The distribution of idiographic accuracy correlations for the judges in Study Two for male, female and all targets in both conditions and the overall sample

Targets	Mean	SD	Minimum	Maximum	Skewness	Kurtosis
Oriented ‘Towards’ Condition (*N* = 29)						
Male Targets Only (k = 11)	.28	.30	-.61	.81	-.75	.19
Female Targets Only (k = 11)	.13	.29	-.37	.78	.57	-.35
All Targets Together (k = 22)	.20	.20	-.30	.64	-.37	.22
Oriented ‘Away’ Condition (*N* = 29)						
Male Targets Only (k = 11)	.28	.31	-.32	.83	-.35	-.81
Female Targets Only (k = 11)	.19	.33	-.57	.81	-.29	.19
All Targets Together (k = 22)	.23	.21	-.20	.57	-.48	-.63
Study Two Overall (*N* = 58)						
Male Targets Only (k = 11)	.36	.35	-.35	.86	-.12	-1.16
Female Targets Only (k = 11)	.14	.28	-.35	.76	.22	-.39
All Targets Together (k = 22)	.24	.20	-.29	.57	-.48	.13

### The Accuracy of Study Two Judges

We treated the data set of Study Two as one group of *N* = 58, and conducted nomothetic and idiographic analyses again. We replicated our Study One findings in both cases. We found that the average threat rating received by each target positively correlated with that target’s trait aggression for the male targets (*r*(11) = .71, *95% CI* [.22, .98], *p* = .022) and the female targets (*r*(11) = .31, *95% CI* [−.31, .90], *p* = .352). Again, these correlations did not meaningfully differ (*Z* = 1.13, *p* = .259). The nomothetic accuracy correction for all targets together was also good; *r*(22) = .43, *95% CI* [−.01, .79], *p* = .043. It is therefore no surprise that there was agreement between the average threat rating received by targets in Study Two with the average threat rating received by targets in Study One (for male targets; *r*(11) = .98, *95% CI* [.74, 1.00], *p* < .001; for female targets; *r*(11) = .98, *95% CI* [.91, .99], *p* < .001; for all targets; *r*(22) = .98, *95% CI* [.95, .99], *p* < .001).

We also tested the idiographic accuracy of the judges in Study Two. We tested the overall accuracy with a one-sample t test and found that the individual’s accuracy for the male targets (*t*(57) = 7.39, *p* < .001, *d* = .97), female targets (*t*(57) = 4.06, *p* < .001, *d* = .53) and all the targets (*t*(57) = 8.83, *p*<. 001, *d* = 1.16) were above chance. This replicated our finding in Study One, that threat ratings can reflect the trait aggression of an approaching person.

### Study One and Study Two Participants together

We tested for differences in accuracy between Study One’s sit-down, computer-screen judgment task and Study Two’s standing-up, distance-presentation judgment task using the idiographic accuracy values. Regardless of laboratory set up, the accuracy of threat judgments for reflecting trait aggression was robust and no differences were found in the threat ratings between the studies for male targets (*t*(117) = 0.63, *p* = .529, *d* = .12), female targets (*t*(117) = 0.64, *p* = .521, *d* = .12) or all targets together (*t*(117) = 1.05, *p* = .298, *d* = .19).

With no difference between conditions we could combine the samples from Study One (*n* = 61) and Study Two (*n* = 58) to tentatively form a meta-sample of *N* = 119. This allows us to report on the general performance of judges across both studies (see Table 3). Unsurprisingly, the combined samples showed notably better accuracy than random performance when using threat judgments to detect the aggression in male targets (*t*(118) = 10.38, *p* < .001, *d* = .95), female targets (*t*(118) = 5.37, *p* < .001, *d* = .49) and all targets together (*t*(118) = 12.02, *p* < .001, *d* = 1.10).

**Table 3:** The distribution of idiographic accuracy correlations for all the judges in Study One and Study Two combined for male, female and all targets. (*N* = 119)

Targets	Mean	SD	Minimum	Maximum	Skewness	Kurtosis
Male Targets Only (k = 11)	.30	.31	-.61	.86	-.41	-.41
Female Targets Only (k = 11)	.14	.29	-.57	.81	.25	-.32
All Targets Together (k = 22)	.22	.20	-.30	.64	-.39	-.16

### Study Two Summary Discussion

Study Two differed from Study One methodologically (with judges standing at a distance from projected stimuli) and included a manipulation to try and affect aggression detection accuracy. Our manipulation did not affect threat judgments, with both conditions having almost identical accuracy performance. This is, in part, due to the strong overall accuracy of judges’ threat judgments. Study Two shows more evidence of judges being better able to recognise the aggression of male targets than Study One (see Table 2).

While accuracy of threat judgments were not affected by the orientation manipulation in Study Two, the accuracy of the judges did replicate the findings of accuracy in Study One. This shows that the accuracy of threat judgments is robust, even when the context is changed. Methodologically and experientially, Study One and Study Two were distinct settings, making the consistency of this result promising for further, more street-typical research.

## General Discussion

In the two studies presented in this paper, we demonstrate and replicate evidence that threat judgments are useful; they can be used to accurately detect the aggression of an approaching person. We find strong accuracy in some of our findings (particularly in the judgments of male targets) and suggest that this is a result of our inclusion of more *Relevant* information to aggression (i.e. body shape [Deaner et al. 2012] and gait [Satchell et al. 2016]) in what we made *Available* for our targets to *Detect* and U*tilize* in their judgments of threat. This increase in judgment accuracy comes with a move toward everyday presentations of stimuli. Whilst it is interesting that judges are accurate judges of aggression when observing brief presentations of faces (Boshyan et al. 2013; Carré et al. 2009; Carré et al. 2010), it is important to note that accuracy is strong when considering how approaching strangers would appear in everyday life. Given the growing literature on how other features of a person are *Relevant* to aggression (Deaner et al. 2012; Satchell et al. 2016), future research could continue to expand the context and ‘everyday-ness’ of threat judgment paradigms.

In both studies, there was also clear evidence that individuals differ in their skill at this task. Those who performed poorly at the task, or even had an inverse relationship between threat ratings and target aggression, should be considered as important as those who performed well. Those who are less accurate at judging aggression are not recognised in aggregate, nomothetic analysis and it could be the case that similar research does not notice these individuals. We also note that the majority of our judges were female and perhaps there would be differences in accuracy between male and female judges of threat. Future research could consider the social and wellbeing consequences of being a poor judge of aggressors, especially as a general ‘fear’ of other people can restrict an individual’s movements (Foster et al. 2014).

The targets of the threat judgments in both of the studies presented here were non-offending young adults (in the same age range as our targets.) It is possible that our findings are bound by our sample and therefore do not reflect trying to predict genuine aggressors in everyday life. This is an issue which further research should explore in more detail by using street based judgments of threat. On the other hand, our judges’ accuracy rates are based on trying to detect differences between highly similar individuals. The ability to discriminate between individuals in the ‘middle of the distribution’ of aggression should not be underrated. Overall, future research should expand the distribution of targets to investigate if the accuracy of judgment is robust over a broader spectrum of individuals.

From the information available in this study, it is not possible to know how much various features of the targets contributed to informing threat judgments and how much the various features related to the target’s trait aggression. Statistical approaches to analysing how qualities of a target may communicate properties of that target do exist and are frequently used. Karelaia and Hogarth (2008) reported that Egon Brunswick’s concept of ‘lens modelling’ (where the qualities of a target are mathematically associated with both the visually salient aspects of a target and the judgments made by a perceiver) has become increasingly popular in human judgment studies. Much like Back et al. (2010) demonstrated that ‘speed and energy of body movement’ is an important communicator of an individuals’ extraversion, future lens models of similar work to this could evaluate the contributions of face shape (Carré et al. 2009; Carré et al. 2010), body shape (Deaner et al. 2012) and gait (Satchell et al. 2016) to communicating aggression in threat judgments.

### Conclusion

Threat judgments are a highly typical form of evaluating an approaching stranger. Here we find that threat judgments are useful in detecting the aggressiveness of that unknown person. This finding was shown to be robust across two different experiments. Whilst the results are based on judging only young adults in an arguably safe, laboratory context, future lens model and ‘street’ research could help us understand the utility of feelings of threat.
